# Rett Syndrome and CDKL5 Deficiency Disorder: From Bench to Clinic

**DOI:** 10.3390/ijms20205098

**Published:** 2019-10-15

**Authors:** Shilpa D. Kadam, Brennan J. Sullivan, Archita Goyal, Mary E. Blue, Constance Smith-Hicks

**Affiliations:** 1 The Hugo Moser Research Institute at Kennedy Krieger, Baltimore, MD 21205, USA; SullivanB@kennedykrieger.org (B.J.S.); GoyalAr@kennedykrieger.org (A.G.); 2 Department of Neurology, Johns Hopkins University School of Medicine, Baltimore, MD 21205, USA; 3 The Solomon H. Snyder Department of Neuroscience, Johns Hopkins University School of Medicine, Baltimore, MD 21205, USA

**Keywords:** glutamate toxicity, interneurons, seizures, sleep, preclinical modeling, clinical trials

## Abstract

Rett syndrome (RTT) and CDKL5 deficiency disorder (CDD) are two rare X-linked developmental brain disorders with overlapping but distinct phenotypic features. This review examines the impact of loss of methyl-CpG-binding protein 2 (MeCP2) and cyclin-dependent kinase-like 5 (CDKL5) on clinical phenotype, deficits in synaptic- and circuit-homeostatic mechanisms, seizures, and sleep. In particular, we compare the overlapping and contrasting features between RTT and CDD in clinic and in preclinical studies. Finally, we discuss lessons learned from recent clinical trials while reviewing the findings from pre-clinical studies.

## 1. Introduction

In the following review we compare the overlapping and contrasting features in Rett syndrome (RTT) and CDKL5 disorder (CDD) in clinic, as well as their respective rodent models. As we now seek to translate preclinical discoveries to the clinic, a forthcoming challenge will be successfully evaluating preclinical studies to pursue for their translational utility. Here we reflect upon lessons learned from recent clinical trials while reviewing the progress in preclinical research. Therefore, this review examines the impact of loss of methyl-CpG-binding protein 2 (MeCP2) and cyclin-dependent kinase-like 5 (CDKL5) on clinical phenotype, epilepsy, circuit homeostasis, sleep impairment, and the insights gained from pre-clinical and clinical studies.

### 1.1. Overlapping but Distinct Clinical Phenotypes for Rett Syndrome and CDD

Rett syndrome (RTT), a neurodevelopmental disorder that primarily affects females is typically characterized by loss of language skills and hand use, impaired or absent gait, dyspraxia, cognitive deficits, stereotyped behaviors, seizures, and autonomic irregularities including respiratory and gastrointestinal (GI) dysfunction and premature osteoporosis and osteopenia [1,2]. Mutations in the X-linked gene encoding *MECP2* (methyl-CpG-binding protein 2) account for 90–95% of the case of classic Rett syndrome (RTT) [3,4] while mutations in the X-linked gene encoding cyclin-dependent kinase-like 5 (CDKL5) account from some cases of atypical RTT that manifest with early refractory epilepsy [5]. CDD has overlapping phenotypic features with RTT including seizures and developmental delays, GI dysfunction, scoliosis, limited or absent speech, and sleep disturbances [5,6,7,8]. However, individuals with CDD exhibit severe developmental delay from birth and seizure onset before the age of 3 months [8,9]. Seizures and sleep disturbances are more common in CDD than in RTT, whereas features of regression and spinal curvature are less common in those with *CDKL5* mutations compared to those with *MECP2* mutations [9,10].

### 1.2. Decreased Brain Volume, Dendritic Arborization, and Spine Density in RTT and CDD

**Figure 1:**
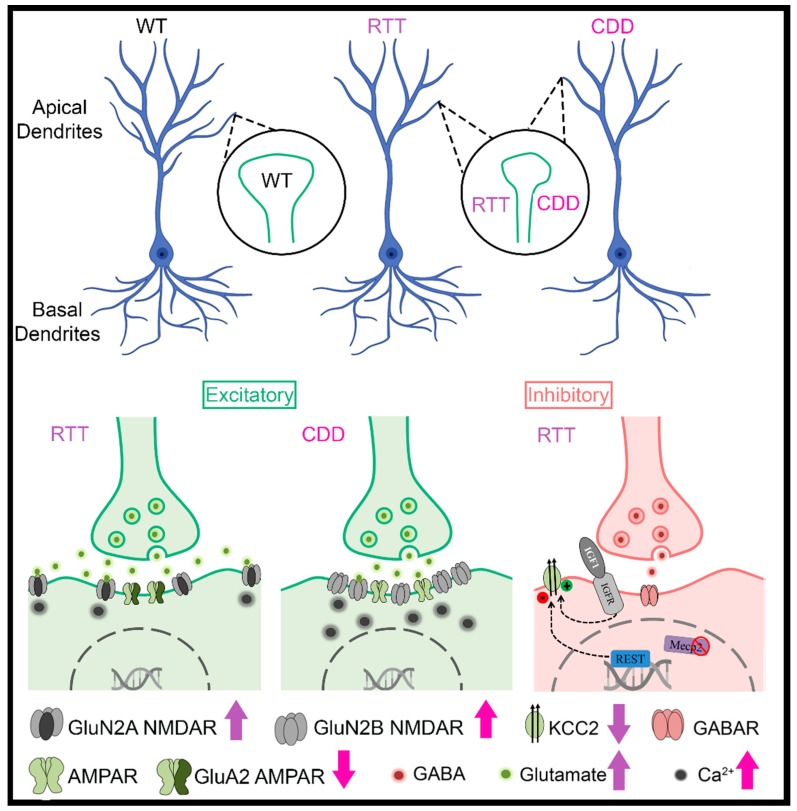
Alterations in dendrites, spines, glutamatergic neurotransmission, and GABAergic neurotransmission are observed in Rett syndrome (RTT) and CDKL5 disorder (CDD) (Schematic generated with the help of Biorender). Arrows indicate deviations from wild-type (WT) controls. (Blue et al., 1999, 2011 [28,29]; Durand et al., 2012 [30]; Johnston et al., 2014 [31]; Lo et al., 2016 [32]; Banerjee et al., 2016 [33]; Della Sala et al., 2016 [24]; Tang et al., 2016, 2019 [34,35]; Okuda et al., 2017 [36]; Tramarin et al., 2018 [37]; Dong et al., 2018 [38]; Zhu and Xiong, 2019 [22]; Ren et al., 2019 [23]; Yennawar et al., 2019 [39]).

One of the defining morphological characteristics of RTT is decreased brain size [11]. MRI studies have shown decreased cross-sectional volumes in selective regions of brains from patients with RTT [12,13,14,15] and in KO mouse models of RTT [16,17,18]. Although fewer MRI studies have been performed on patients with CDD than those with RTT, mild frontal lobe atrophy has been noted [19]. The reductions in cortical volume are consistent with reductions in dendritic arborization and spine density in postmortem brains from patients with RTT and in KO mouse models of RTT [17,20,21] (Figure 1). Likewise, decreases in dendritic arborization [22,23], reductions in spine density, PSD-95-positive synaptic puncta, and alterations in spine morphology [23,24,25] have been noted in CDKL5 KO mouse models (Figure 1). In addition, dysfunction in dendritic spine formation is evident in both CDKL5 mutations associated with impaired PSD-95 interactions [26] and in RTT via disruption of the DiGeorge syndrome critical region 8 (DGCR8) complex, a critical component of the nuclear microRNA-processing machinery [27].

### 1.3. Seizures

Epilepsy is a major co-morbidity in RTT (60–80%) [1,40,41,42], and clinical studies show that early-onset epilepsy portends poorer prognosis with more severe regression [40,43,44]. In CDD, early infantile onset refractory epilepsy is typical and ninety percent of CDD patients have seizure onsets between six weeks to three months of age with 80% percent having daily seizures [45,46]. The precise mechanisms by which loss of MeCP2 function in RTT and CDKL5 in CDD result in epilepsy remain unclear. Hyperexcitability in seizure syndromes is thought to result from an imbalance of inhibition and excitation. Counter-intuitively, enhanced inhibition can also lead to hyperexcitability and seizure activity [47]. Evidence exists for alterations in both excitatory (glutamatergic) and inhibitory (GABAergic) mechanisms that result in the excitatory/inhibitory balance in both RTT and CDD. Seizure severity and management differs by age in both RTT and CDD. RTT is divided by four stages: Stage I: Early onset between 6–18 months, Stage II: Rapid deterioration between ages 1 to 4 yrs., Stage III: Plateau between the ages of 2 to 10 years and can last for many years, and Stage IV: Associated with late motor deterioration [1,2]. The percentage of girls with RTT experiencing seizures increases with age from 33% at 3–5 years to 84% at 15–30 years of age [40]. Seizures in girls within the age group from 10–14 years were reported to be most difficult to treat, requiring a mean of three different anti-epileptic drugs [48]. In contrast, CDD, with its early onset of seizures fits an infantile epileptic encephalopathy profile [6]. These temporal stages may indicate an age-dependent susceptibility to circuit excitability underlying spontaneous seizures in RTT and CDD.

### 1.4. Glutamatergic Alterations in RTT and CDD

Alterations in cortical glutamatergic synaptic responses and excitatory connectivity play important roles in RTT but these studies vary in the degree to which the balance between excitation and inhibition is shifted in favor of inhibition [32,49] or hyperexcitability [50,51]. The differences in study results may be due to age and region-specific differences in glutamate receptor expression. Our previous autoradiographic studies in postmortem samples from patients with RTT and in presymptomatic *Mecp2*-null mice showed a biphasic pattern of expression of ionotropic glutamate N-methyl-D-aspartate receptor (NMDAR) in frontal cortex such that densities of NMDARs were higher than normal controls at young ages but lower than normal at older stages [28,29]. In primary somatosensory cortex of *Mecp2*-null mice, extrasynaptic NMDA receptor-mediated responses are increased, and administration of the NMDAR antagonist memantine blocks extrasynaptic NMDA receptor-mediated responses [32]. These results indicate over-activation of excitatory mechanisms in young animals that could lead to “burn out” of excitatory circuits at older stages where the balance is shifted to inhibition. This notion is consistent with results from primary neuronal cultures studies that show that the loss of MeCP2 increases the susceptibility of neurons to excitotoxicity [52,53]. Early-life seizures produce a variety of cellular and molecular changes in the developing RTT brain [54,55,56,57] that may further disease progression.

CDKL5 has been implicated in neuronal survival via SMAD3 signaling suggesting neuronal susceptibility to excitotoxic injury in CDD [58]. Studies have shown that CDKL5 deficiency in primary hippocampal neurons leads to dysregulated expression of the alpha-amino-3-hydroxy-5-methyl-4-iso-xazole propionic acid receptors (AMPARs), particularly the GluA2 subunits [37]. A skew towards GluA2-lacking AMPARs with calcium permeability could significantly increase excitability and affect synaptic function. The findings of increased expression of GluN2B in postsynaptic densities in the hippocampus of *Cdkl5*^(−/y)^ mice may also contribute to enhanced seizure susceptibility [36].

### 1.5. Impaired Maturation of the GABAergic System

Other studies propose RTT as a disease involving interneuronopathy [59]. *Mecp2* mutations perturb postnatal maturation of the connectivity, function, and plasticity of inhibitory GABAergic interneurons in symptomatic *Mecp2*-null mice [60,61]. The development of GABAergic circuits is a prolonged process that begins during mid-gestation and is not complete until the end of adolescence. The transcription factor Dlx5, one of the factors regulating the differentiation and maturation of forebrain GABAergic interneurons, has been identified as a direct target of MeCP2 [62,63]. Temporal- and location-specific investigation of alterations in the expression of γ-amino butyric acid transporter 1, vesicular GABA transporter, and glutamic acid decarboxylase 67kD showed significant location-specific down-regulation of synaptic GABA transporters in *Mecp2*-null brains with unaltered densities of GAD67-positive interneurons [64], highlighting the synaptic pathophysiology associated with loss of MeCP2.

Multiple studies indicate that MeCP2 expression in parvalbumin-expressing GABAergic (PV+) cells during the critical period is essential for local circuit functions that underlie circuit formation and experience-dependent cortical plasticity mechanisms. In one study, specific deletion of *Mecp2* in PV+ and somatostatin (SOM)+ neurons had distinct effects in which mice lacking MeCP2 in PV+ neurons developed motor, sensory, memory, and social deficits, while those lacking MeCP2 in SOM+ neurons exhibited seizures and stereotypies [65]. In another study, selective loss of MeCP2 in GABAergic SOM+ cells did not affect the critical period plasticity in primary visual cortex (V1) but MeCP2-lacking PV+ cells exhibited high intrinsic excitability, selectively reduced efficacy of recurrent excitatory synapses in layer 4 circuits, and decreased evoked visual responses [66]. Others have shown that symptomatic male *Mecp2*-null and female *Mecp2*-heterozygous mice have elevated PV expression in both somatosensory (SI) and motor (M1) cortices together with excessive excitatory inputs converging onto PV expressing interneurons [67]. These changes lead to reduced amplitude and spatial spread of synaptically induced neuronal depolarization in S1 and impaired motor learning-dependent changes of PV expression and structural synaptic plasticity.

Impairments in PV interneuron circuits also are implicated in defects in visual processing and visual cortical plasticity in both RTT and CDD [68,69]. *Mecp2*-null and *Mecp2*-heterozygous female mice and RTT patients exhibit a comparable decrease in visually evoked potential amplitudes especially in the later stages of the disorder associated with lower visual spatial acuity [30,70,71]. Interestingly, the density of PV+ but not other types of interneurons is higher in V1 of *Mecp2*-null mice; these PV+ interneurons are hyper-connected with pyramidal neurons [72]. Deletion of NR2A receptors in *Mecp2*-null mice restored cortical processing and connectivity and prevented the onset of visual deficits [71], suggesting an impairment in the E/I balance in the visual cortex that may mediate the altered visual responses in RTT.

The visual cortex has a well-defined critical window necessary for proper development, and in *Mecp2-*null mice this critical period closes prematurely [60]. This altered critical period is linked to an increase in GABA synthetic enzymes, vesicular GABA transporter, perineuronal nets (PNNs), and enhanced GABA transmission among PV+ interneurons [60,72]. NMDAR maturation (NR2B to NR2A subunit switch) is accelerated in PV+ interneurons but slower in pyramidal cells within V1 of *Mecp2*-null compared to age-matched wild-type (WT) mice [73].

Visual attention deficits and reduced visual acuity are also common in CDD. Studies investigating the visual cortex in murine models of CDD have elicited an impaired developmental trajectory of PV+ interneurons, represented by fainter appearance of perineuronal nets at the closure of the critical period and [74]. In addition, c-Fos expression in V1 was reduced markedly, indicative of hypoactive circuits [74]. However, glutamatergic presynaptic structures were increased, while postsynaptic PSD-95 and Homer were significantly down regulated. As with *Mecp2*-null mice, the density of PV+ interneurons was higher in V1 of *Cdkl5*^(−/y)^ mice. Mutants showed reduced density and altered morphology of spines, decreased excitatory synapse marker PSD-95 in the dorsal Lateral Geniculate Nucleus (dLGN) and in V1, and an increase in the inhibitory marker VGAT in V1 [25]. However, the organization of the retinal circuits was not altered [25]. Taken together the results suggest that disruption in the cellular and synaptic organization of dLGN and V1 as well as the shift in the balance of EI underlie the visual deficits observed in CDD [25,74].

The interneuron cell-type and circuit specificity for abnormal densities and innervation properties reported highlight the regionality of RTT- and CDD-related pathology. Different circuits in the brain have different critical windows for maturation. The integration of interneurons with local and long-distance innervation occurs over extended periods of time in both rodents and humans and therefore could be affected differentially in RTT and CDD and temporally advancing ages. The loss of the E/I balance associated with interneuron dysfunction is known to underlie both learning disabilities and autism spectrum disorders [75,76].

### 1.6. The Emerging Role of Astrocytes

Astrocytes play a major role in the pathogenesis and abnormal neurodevelopment underlying RTT [77,78,79]. Astrocytes actively control dendritic growth, synaptogenesis, synapse number, synapse function, and synaptic plasticity [80] and these processes are disrupted in *Mecp2*-null mice [77,78,79]. Abnormal astrocytic calcium homeostasis and excessive activation of extrasynaptic NMDARs caused by *Mecp2* deletion in astrocytes in vivo indicate the role of astrocytes in the excessive excitation in and around synapses in RTT [38]. Further, astrocytes buffer extracellular potassium concentrations in the synaptic cleft. Kir4.1 is an inwardly rectifying potassium channel that is responsible for a majority of the astrocytic potassium buffering [81]. Astrocytes from MeCP2-deficient mice express low level of Kir4.1 and subsequent elevated levels of extracellular potassium concentration [82], suggesting a role for astrocytic MeCP2 in the regulation of neuronal excitability [82]. Due to their peri-synaptic location, astrocytes could drive the elevated release of glutamate detected in RTT [31,79] modulating functional gamma oscillations known to underlie cognitive behavior [83]. In fully symptomatic MeCP2–308 male mice, region-specific astrocyte atrophy has been identified [84]. Activation of RhoGTPases, which regulate actin cytoskeleton dynamics and are crucial in neuronal structural synaptic plasticity, by the bacterial cytotoxic necrotizing factor 1 (CNF1), improved RTT-related behavioral impairments and dramatically reversed the astrocytic atrophy in *Mecp2*–308 mice [85]. Further, in *Mecp2*–308 heterozygous female mice, CNF1 treatment rescues RTT-related behavioral impairments, functional synaptic plasticity, and brain mitochondrial homeostasis [86]. These results suggest that mitochondrial dysfunction and abnormal RhoGTPase activation can disrupt crucial synaptic plasticity and are associated with astrocytic atrophy. Altered RhoGTPase signaling has also been implicated in mouse models of CDD [87].

### 1.7. Circuit Homeostasis in RTT

Understanding neurophysiological correlates underlying impaired intellectual disabilities in genetic disorders is one of the frontiers of neuroscience. Recent research has highlighted the complementary mechanisms by which MeCP2 deficits hamper inhibition via two dueling mechanisms. One mechanism involved reducing responses of PV+ interneurons and another by altering the polarity of GABAergic inhibition [33]. This dual defect would not only lead to hyperexcitable cortical circuits but also circuits that are unable to respond effectively to incoming cues of behavioral states or sensorimotor stimuli. The behavioral-dependent homeostatic function of PV+ interneurons is also critical in the regulation of cortico-cortical and striato-cortical circuit gamma oscillations [88] that underlie cortical engagement during exploration and novel activity. Recent work has shown that MeCP2 deficiency impairs the long-range connectivity of new neurons born in the adult hippocampus to the cortex, whereas their connectivity within the local hippocampal circuits or within subcortical regions is not significantly affected [89]. Similarly, event-related deficits in *Cdkl5*^−/y^ mice suggest impairments in long-range communication [90].

Another potential mechanism by which MeCP2 deficits could manifest their effects on circuit development is through effects on the electroneutral cation-Cl- cotransporter KCC2 (K^+^/Cl^−^ exporter), the chief Cl^−^ exporter in neurons. Fast synaptic GABAergic inhibition relies on the ability for neurons to tightly regulate intracellular Cl- [91]. KCC2 hypofunction causes neuronal Cl- dysregulation and results in inefficacious synaptic inhibition that mediates ineffective neural coding and runaway excitation [91]. *Mecp2*-null mice have reduced expression of KCC2 [35] (Figure 1). Perforated patch recordings in V1 have demonstrated a depolarized reversal potential for GABA in mutant mice compared to WT. Hyperpolarized values for the GABA reversal potential were restored by the NKCC1 inhibitor bumetanide, as well as by recombinant human insulin-like growth factor-1 [33]. Further, recent research shows a therapeutic effect for other pharmacological agents that enhance KCC2 gene expression in both human iPSC neurons from RTT patients and in *Mecp2*-mutant mice [34], suggesting that targeting this mechanism may have important clinical ramifications.

### 1.8. Sleep in RTT and in CDD

Sleep dysfunction is a prominent co-morbidity reported in 80% of patients with RTT [92,93,94], and sleep deprivation has been investigated as an aggravating factor in epilepsy [95]. Low sleep efficiency, long sleep-onset latency, and a short and fragmented total sleep time have been reported in RTT [94]. Studies using mice with different *Mecp2* mutations have identified several impairments that likely contribute to the pathophysiology of the sleep disorder [96,97]. In patients with RTT, examinations of the trajectory of the sleep dysfunction over a 12-year period found the rate of occurrence to be 80% and that the severity of the sleep disruption decreased with age [93]. Groups with high- or low-baseline prevalence of sleep disturbances like night laughing or night screaming were identified and those with larger deletions in *MECP2* were often in the high-prevalence group [93].

The sleep structure of *Mecp2*-null mice is altered and shows longer wake-cycles that were associated with poor quality of slow-wave sleep and significant increases in in vivo cortical glutamate loads compared to wild-type mice [31]. Documentation of similar glutamate loads in girls with RTT [98] and significantly altered slow-wave sleep patterns [99] bolster the pre-clinical research findings. Interestingly, a qEEG study showed heightened delta power during NREM sleep in girls ranging from 1 to 9 years of age [99]. This pattern was more apparent in younger girls with RTT (age 2–5) and was associated with a persistence of high gamma in the occipital leads. Gamma power in age-matched control groups showed a significant decrease in gamma power from age group 2–5 years to 6–9 years of age in occipital leads. These findings are consistent with the report of higher expression of glutamate receptors in younger (i.e., ≤8 years old) RTT brains [100] as well as in two-week-old *Mecp2*-null male mice (*Mecp2tm1.1Bird*) [28]. Similar qEEG studies in patients with CDD-related sleep disorders are needed. Since sleep states are associated with significant synaptic homeostatic scaling (Diering et al., 2017), the loss of the glutamate homeostasis in RTT [31] may indicate a role of increased excitation in the underlying sleep dysfunction. Likewise, in CDD, which is now identified as an independent entity, there are reports of a higher prevalence of sleep disturbances with males being affected more severely than females [101]. Additionally, sleep apneas have been documented in both patients and the mouse model of CDD [102,103]. Finally, disruptions in the circadian rhythms have been reported in a mouse model of CDD [87].

### 1.9. Insights from Pre-Clinical Studies Targeting Glutamatergic Pathways

Many excellent reviews of pre-clinical studies exist that examine several mechanisms that are dysregulated in RTT [104,105,106,107,108]. Here we focus on mechanisms that target the glutamate pathway and shed insight into therapeutic strategies for RTT. MeCP2 modulates glutamate activity by transcriptional repression and epigenetic modification of ionotropic and metabotropic receptors. We have demonstrated altered expression of the ionotropic glutamate NMDARs in postmortem samples from patients with RTT and *Mecp2*-null mice [28,29], while others show increased levels of glutamate in patients with RTT [109] and the RTT mouse model [31]. Genetic deletion of the GluN2A subunit of the NMDAR in *Mecp2*-null mice prevents loss of cortical function and visual cortical activity [30] (Figure 1). Consistent with these findings, acute and chronic administration of ketamine, an NMDAR non-competitive antagonist, improves life span, motor coordination, and visual motor processing [71,110], while memantine, a noncompetitive NMDAR antagonist, produces a partial reversal in synaptic deficits in vitro without improvement in disease severity or progression in *Mecp2*-null mice [111].

Similar to the aberrant expression of ionotropic glutamate receptors, reductions in metabotropic glutamate receptor-5 (mGluR5) protein levels have been reported in both the motor cortex from RTT autopsy samples and in the brain of mouse models of RTT, implicating mGluR5 in the pathogenesis of RTT [112]. In addition, mGluR-5 protein-synthesis-dependent synaptic plasticity is attenuated in MeCP2- Schaffer collateral SC-CA1 synapses [112,113]. Tao and colleagues hypothesized that loss of the transcriptional repressor activity of MeCP2 leads to translational dysregulation of mRNAs that are tightly regulated by the mGluR5 pathway [113]. Consistent with this hypothesis they showed that a subset of ribosome-bound mRNAs that overlapped with FMRP direct targets and autism susceptibility genes were aberrantly up-regulated in hippocampal CA1 neurons of *Mecp2*-null mice. Intriguingly, chronic treatment of *Mecp2*-null mice with an mGluR5-negative allosteric modulator down regulated the ribosome-bound mRNAs and improved the life span and hippocampal cell size but had no impact on motor or anxiety deficits [113]. VU0462807, a positive allosteric modulator of mGluR5, rescued the synaptic plasticity deficits, and motor function, notably open field behavior and gait, reduced repetitive clasping and normalized deficits in cued-fear conditioning without increasing the risk for epilepsy or adversely impacting cardiorespiratory function in the mice [112].

In addition to its role in transcriptional repression of metabotropic glutamate receptors, MeCP2 epigenetically regulates the expression of the metabotropic glutamate receptor 7 (mGlu7), at presynaptic GABAergic synapses where mGlu7 acts to decrease neurotransmitter release and is necessary for induction of LTP at Schaffer collateral (SC)-CA1 synapses [114]. The loss of MeCP2 in GABAergic interneurons is sufficient to recapitulate several phenotypes of RTT and mGlu7 RNA, protein expression is reduced in rodent models of RTT syndrome, and SC-CA1 LTP is impaired in male and female mouse models of MeCP2 deficiency [115]. The likelihood that the reduction in mGlu7 contributes to the RTT phenotype was further supported by the reduction of mGlu7 protein levels in the motor cortex of human RTT autopsy samples [115]. To test this hypothesis, *Mecp2*-null mice were treated with positive allosteric modulators of mGlu7, and the core phenotypes of contextual fear memory, social recognition, and apneas were reversed in the mouse model of RTT [115].

Taken together these results suggest that targeting both metabotropic and ionotropic glutamate receptor pathways may be a viable therapeutic approach to treating cognitive and respiratory deficits in RTT. In addition, these results highlight the challenges of correcting the widespread core deficits of RTT and suggest that an approach that targets multiple mechanisms is needed.

Differences in glutamate-mediated plasticity mechanisms are also noted in mouse models of CDD, where expression of AMPARs lacking the GluA2 subunit is increased [37] (Figure 1). A similar trend is observed in human autopsy brain samples from individuals with CDD. Acute treatment with IEM-1460, a putative GluA2-lacking AMPAR antagonist, normalized social behavior and improved working memory in a mouse model of CDD [39].

## 2. Current Clinical Trials

### 2.1. Rett Syndrome

Several phase 2 clinical trials are recruiting RTT patients in 2019 (https://clinicaltrial.gov). These studies are designed to evaluate the efficacy of the following compounds: Ketamine, ANAVEX2–73, cannabidiol, triheptanoin, and Trofinetide and are well supported by preclinical studies. Of these, ketamine, a non-competitive NMDAR antagonist; cannabidiol, which acts through 5HT1A and glutamate receptors [116]; and ANAVEX2–73, a mixed muscarinic and *σ*_1_receptor agonist [117], are compounds with direct and indirect actions on glutamatergic pathways. Triheptanoin is used to improve energy generation via the Kreb cycle [118], and Trofinetide, a tripeptide of insulin-like growth factor 1, a growth factor involved in brain development and plasticity [119], have no known involvement with the glutamate pathway.

These studies target different mechanisms of action and different clinical features of Rett. The ANAVEX2–73 trial is open to individuals aged 18 years and over who are diagnosed with classic Rett and examines the effect of ANAVEX2–73 on behavior and mood as well as seizure and sleep. GW Research sponsors the cannabidiol study. It is a randomized, placebo-controlled parallel assignment study that is open to individuals from 2–18 years old and examines the dose effect on behavioral, sleep, and motor features. A crossover study to assess the safety, tolerability, and efficacy of oral ketamine in Rett syndrome females between the ages of 6–12 years old is currently underway. Although the primary outcome measure is safety, several exploratory measures are being pursued to evaluate behavior, sleep, motor function, and quality of life. Mitochondrial dysfunction manifesting in patients with Rett syndrome, dyskinesia, and epilepsy is being targeted with Triheptanoin in an open-label study sponsored by Ultragenyx and the Rett Syndrome Research Trust. This study is open to individuals with classic Rett who are aged 2 years and older. The primary outcome measures are the frequency of seizures and dystonia while secondary outcome measures examine several features of dystonia, pain, functional mobility, and mood. A double-blind placebo-controlled exploratory phase 2 trial of Trofinetide in RTT indicated that Trofinetide was well tolerated and was safe for use in adolescent and adults females with Rett syndrome [120]. Although this study was of short duration and only included a small number of individuals (56 females with RTT), both caregivers and clinicians noted clinically meaningful improvements [120]. A follow-up study of 82 patients confirmed initial findings of clinically relevant improvements on the Rett syndrome behavior questionnaire and other behaviorally relevant scales [121]. In light of these encouraging findings, a phase 3 Trofinetide study is being planned but is not yet recruiting participants.

The above-mentioned studies have varying designs—open label, randomized double-blind, placebo-controlled with open-label extension, parallel assignment, and crossover studies. These designs were likely utilized to reduce the effects of small size of the available pool of affected individuals. Of note, the cannabidiol trial is the largest study to date and targets 252 participants. The remaining studies target fewer than 50 individuals per trial.

### 2.2. CDKL5 Deficiency Disorder

Patients with CDD are currently being recruited to participate in one of four clinical trials: Ganaxolone, Ataluren, fenfluramine, and TAK-935/OV-935 (https://clinicaltrials.gov). These trials are motivated by an understanding of the biology of the disorders without prior pre-clinical studies in rodent models of CDD. Ganaxolone is a high-affinity allosteric modulator of GABA_A_Rs that acts to restore the balance of excitation and inhibition [122,123]. The Ganaxolone/Marigold Study (Marinus) is a double-blind, placebo-controlled phase 3 trial that examines the effect of Ganaxolone on seizures, behavior, and sleep in patients between the ages of 2 and 21. These endpoints proved useful in previous non-CDD clinical studies with Ganaxolone. PTC Therapeutics, a NYU-based company, has initiated a phase 2 crossover study of Ataluren for the treatment of drug-resistant epilepsy in patients with nonsense mutations with CDD. There has been some concern that Ataluren does not effectively cross the blood–brain barrier; however, a proof of principle study indicated that at higher doses Ataluren breaches the blood–brain barrier in the infantile neuronal ceroid lipofucinosis mouse model, where it increases the lysosomal enzyme palmitoyl-protein thioesterase 1 (PPT1) enzyme activity and protein levels [124]. The fenfluramine ZX008 study (NYU, Zogenix) is an open-label trial of fenfluramine for treatment of seizures in patients with CDD. Fenfluramine was shown to be effective in open-label studies of Dravet syndrome and zebra fish model of Dravet syndrome [125,126] and is being considered for its utility in CDD. Fenfluramine has a novel mechanism of action that includes multiple receptors. It is shown to act both on the release and inhibition of serotonin reuptake as well as a positive allosteric modulator at the σ-1 receptor that is involved in modulation of glutamate [127]. The final agent TAK-935 (Takeda, Ovid) is a potent, highly selective inhibitor of the enzyme cholesterol 24-hydroxylase CH24H that is being used in the study of rare pediatric epilepsies. CH24H is predominantly expressed in the brain, where it plays a central role in cholesterol homeostasis. CH24H converts cholesterol to 24-S-hydroxycholesterol(24HC) which then exits the brain into the blood plasma circulation. CH24H is involved in over-activation of the glutamatergic pathway through modulation of the NMDA channel [128,129], thus suggesting a potential role in epilepsy.

### 2.3. Lessons Learned from Clinical Trials

Clinical trials in Rett syndrome and CDD have multiple limitations, including a relatively small pool of affected individuals and variability in clinical phenotype and disease progression. To circumvent challenges associated with the lack of eligible study participants, researchers initially opted to use open-label studies, or crossover trial designs, although both have had their share of challenges. The placebo effect, a common confounder in open-label studies was reported to be about 63% in one trial [130]. In a natural history study of RTT patients examined from 2006–2015, the number of patients designated as having classic RTT accounted for 76% of the 1205 individuals who enrolled, while 6% had atypical RTT with a mild phenotype and 6% had atypical RTT with a severe phenotype [10]. The percentages of patients who were seizure-free in a six-month period were 68, 84, and 66 respectively, while the remainder of patients reported to have seizures ranging from several per day to once per month [10]. This variability in clinical seizures is expected to impact trials that are designed to occur over longer periods of time. More recently, trials are designed to be double-blind placebo-controlled with an open-label period. The effectiveness of this design is yet to be determined but it is thought to be a good compromise. Variability in the age at which regression occurs and the definition of regression [131,132,133], are other challenges that have been addressed by including patients that are older than 5 years old as they are thought to be outside of the period of regression [134] (http://clinicaltrials.gov).

To date, two clinical trials using dextromethorphan for girls with Rett syndrome have been completed. Safety determination, dose exploration, and an evaluation of outcomes were completed in an open-label study that showed encouraging treatment effect on language [135]. Consequently, a double-blind placebo control study was performed. The baseline language values in the placebo group were significantly higher than in the treatment group, suggesting an imbalanced allocation of language abilities between groups (Smith-Hicks, personal communication). Over representation of mild baseline phenotype in the treatment or placebo group confounds the results of treatment trials and highlights the need for objective measures that can be used for stratification and thus allow for a more equal distribution of clinical phenotypes between treatment arms. The lack of valid outcome measures in non-verbal individuals with cognitive impairment and limited hand use continues to be an area of concern. However, efforts to develop appropriate outcome measures are being actively pursued [70,136]. Other trials assessing the efficacy of NMDAR antagonists include an open-label ketamine trial that was terminated before completion and a ketamine placebo-controlled trial that is currently recruiting study participants (https://clinicaltrials.gov).

While clinical trial readiness is further along in RTT and the participant pool is larger in RTT when compared to CDD, progress in both disorders will continue to require large scale cooperative efforts between international patient groups, scientists, physicians, industry and funding organizations.

## 3. Conclusions

As we now seek to translate preclinical discoveries to the clinic, a forthcoming challenge will be successfully evaluating preclinical studies to pursue for their translational utility. The research findings reviewed here have identified several neuronal and non-neuronal pathways underlying both RTT and CDD neuropathology. The wide range of impairments are associated with cell-type, circuit, age, and model specific alterations. Most of the studies presented here have used *Mecp2*-null male mice or rats as their phenotype mirrors the severity of RTT albeit that RTT is not typically lethal as is observed in most KO models, and the developmental onset of symptoms is later than is observed in RTT. Fewer studies have examined *Mecp2*-heterozygous models, which matches the genotype of girls with RTT, as the phenotypic features develop much later and are less severe than is experienced in patients with RTT. In any case, all the pre-clinical findings have the caveat of not being fully relevant for translation to female patients with RTT. For CDD, the current clinical trials do not stem from any specific pre-clinical findings. CDD has only recently been defined as a discrete disorder from RTT [9] and much less is known about its role in development, and there are fewer studies in CDKL5-deficient animal models. However, this may change given the recent rise in CDD-related preclinical research. CDD clinical trials will likely be underpowered for sample size unless strong international collaboration for patient recruitment is established.
